# Comparative Pharmacokinetic Study of Forchlorfenuron in Adult and Juvenile Rats

**DOI:** 10.3390/molecules26144276

**Published:** 2021-07-14

**Authors:** Li Ping, Bingyong Xu, Qian Zhou, Yawen Hong, Qingmei Sun, Jincheng Wang, Difeng Zhu

**Affiliations:** 1 Center for Drug Safety Evaluation and Research, College of Pharmaceutical Sciences, Zhejiang University, Hangzhou 310058, China; pingli552@zju.edu.cn (L.P.); hongyawen61@zju.edu.cn (Y.H.); 15658223676@163.com (Q.S.); 2 Hangzhou Heze Pharmaceutical Technology Co., Ltd., Hangzhou 310018, China; xubingyong@hezepharm.com; 3 Department of Pharmacy, Hangzhou Medical College, Hangzhou 310053, China; qianz1220@hmc.edu.cn

**Keywords:** pharmacokinetics, forchlorfenuron, juvenile rat, liquid chromatography-mass spectrometry

## Abstract

Forchlorfenuron (CPPU) is a plant growth regulator extensively used in agriculture. However, studies on CPPU pharmacokinetics are lacking. We established and validated a rapid, sensitive, and accurate liquid chromatography–mass spectrometry method for CPPU detection in rat plasma. CPPU pharmacokinetics was evaluated in adult and juvenile rats orally treated with 10, 30, and 90 mg/kg of the compound. The area under the plasma drug concentration–time curve from 0 to 24 h (AUC), at the final time point sampled (AUC_0–t_), and the maximum drug concentration of CPPU increased in a dose-dependent manner. The pharmacokinetic parameters AUC_0–t_ and absolute bioavailability were higher in the juvenile rats than in adult rats. The mean residence time and AUC_0–t_ of juvenile rats in the gavage groups, except for the 10 mg/kg dose, were significantly higher in comparison to those observed for adult rats (*p* < 0.001). The plasma clearance of CPPU in juvenile rats was slightly lower than that in the adult rats. Taken together, juvenile rats were more sensitive to CPPU than adult rats, which indicates potential safety risks of CPPU in minors.

## 1. Introduction

Forchlorfenuron, also known as 1-(2-chloro-4-pyridyl)-3-phenylurea (CPPU), is a synthetic phenyl urea-derived cytokinin extensively used as a plant growth regulator in agriculture. Its major effects include the promotion of cell division, growth, and differentiation. CPPU has already been registered for use in many countries, including European Union countries, the USA, and China [1], and is widely applied in kiwifruit, watermelon, cucumber, and grape cultivation [2]. However, the excessive and inappropriate use of CPPU in recent years has led to concerns regarding its safety [3].

Previous research has reported CPPU-associated toxicity. Gong et al. found that CPPU could induce cardiotoxicity, including cardiac deformation, cardiac contractile dysfunction, and anemia, in zebrafish [4]. An in vitro study demonstrated that CPPU was cytotoxic to normal Chinese hamster ovary cells, with an IC_50_ value of 12.1 ± 2.1 μM [5]. A study by Qian et al. showed that CPPU down-regulated the expression of CYP17A1 and CYP19A1, and, consequently, estradiol and progesterone production in Sprague-Dawley rats [6], while also suggesting that it may promote estradiol secretion. Adverse effects have been reported in prepubertal female rats [7], indicative of reproductive toxicity following CPPU exposure. CYP17A1 and CYP19A1 are also expressed in humans, with CYP17A1 playing a key role in steroid hormone synthesis and CYP19A1 being the rate-limiting enzyme of estrogen synthesis from androgen [8,9].

A previous study demonstrated that juvenile animals are more susceptible to the acute effects of some organophosphorus insecticides than adults [10]. Based on these reports, pesticide exposure is expected to induce a more toxic response in children. The risk of pesticide exposure in children has received widespread attention in the field of public health. In this context, children should not be considered small adults, but rather a unique subpopulation with an altered vulnerability to chemical insults due to differences in physiological and metabolic functions [11,12].

CPPU residues have been reported in fruit [2,13], suggesting that humans may be exposed to CPPU through the intake of both fruit and vegetables [14,15]. While CPPU ingestion may have detrimental health effects, studies on its pharmacokinetics (PK) in animals have been limited, it is necessary to develop a method for the detection of plasma CPPU. The PK characteristics of CPPU in children are different from those in adults, as the former are more sensitive to chemical compounds [12,16,17,18]. While the PK characteristics of CPPU in juvenile rats are currently unclear, understanding these may provide a basis for the further study of pesticide exposure in children. Therefore, it is important to explore the comparative PK characteristics of CPPU in juvenile versus adult rats.

In this study, we developed and validated a rapid liquid chromatography–mass spectrometry (LC-MS/MS) method to examine CPPU concentration in rat plasma. We applied the method to compare the PK characteristics of CPPU in adult and juvenile rats. Our findings provide evidence for the risks of CPPU exposure in children as well as a scientific basis for the understanding of its metabolism in humans.

## 2. Results and Discussion

### 2.1. Optimization of Sample Preparation

To remove proteins and other components that could potentially cause interference, protein precipitation and liquid-liquid extraction methods were performed, while keeping in mind the chemical properties of CPPU. Both methods indicated that direct protein precipitation with methanol produced a better separation and extraction recovery rate. Thus, direct protein precipitation with methanol was selected for sample preparation.

### 2.2. Optimization of LC and MS Conditions

To optimize the conditions for the detection of CPPU and curcumin, the internal standard (IS), different chromatography and mass spectrometry conditions were evaluated. We compared the results obtained using Xbridge™ C18 (2.1 mm × 50 mm, 3.5 μm; Waters, Milford, MA, USA) column and the ACQUITYUPLCBEHC18 (2.1 mm × 50 mm, 1.7 μm; Waters) column. The latter exhibited a better resolution and peak shape of the analyte and was therefore chosen for further analysis. Acetonitrile/water and methanol/water binary solvent system using different buffers, such as ammonium formate and formic acid, were tested for the mobile phase. Acetonitrile/water containing 0.1% formic acid was finally selected, where gradient elution could improve peak shape symmetry and enhance the signal. Finally, we examined flow rates in the range of 0.2–0.5 mL/min and found that 0.3 mL/min was the best condition.

**Figure 1:**
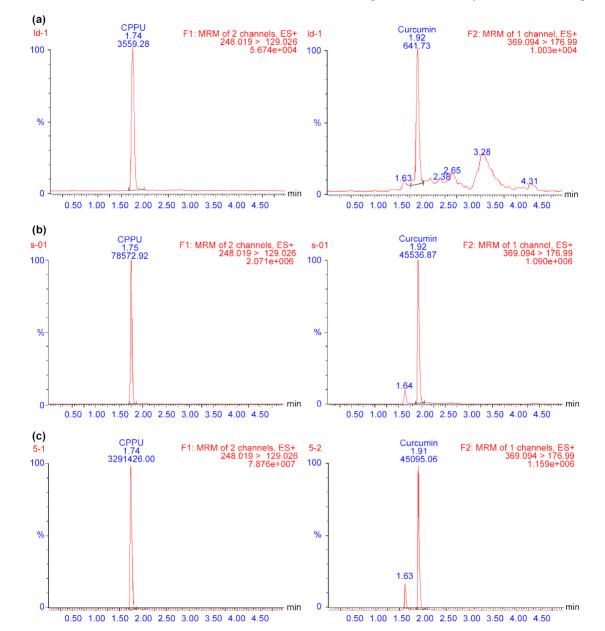
Typical LC/MS-MS chromatograms of (**a**) blank plasma, (**b**) blank plasma spiked with CPPU at the LLOQ, and (**c**) actual sample obtained from a rat 0.5 h after intragastric administration of CPPU. CPPU—1-(2-chloro-4-pyridyl)-3-phenylurea, forchlorfenuron; LC/MS-MS—liquid chromatography-mass spectrometry; LLOQ—lower limit of quantification.

Electrospray ionization (ESI) was used to generate ions due to its high sensitivity and fragmentation. To optimize the ESI conditions for detecting CPPU and IS, positive (ESI+) and negative (ESI−) ion detection modes were explored. Both compounds exhibited good responses in the ESI+ detection mode, with a low background noise level. Thus, detection was operated in ESI+ mode. Next, autotune was used to obtain the initial optimal MS parameters; however, the sensitivity for the analyte was not sufficient. Finally, optimal MS conditions, including collision energy, cone voltage, ion source temperature, and desolvation gas temperature, were further optimized via manual manipulation in fine-tuning mode. Representative CPPU LC/MS-MS chromatograms are shown in Figure 1. There was no distinct interference from endogenous peaks under the chromatographic conditions, and the retention times of CPPU and IS were 1.74 and 1.91 min, respectively. These results indicate that the method is specific for the analysis of CPPU in rat plasma.

### 2.3. Method Validation

The CPPU calibration plots were linear in the range of 20–2000 ng/mL using 1/x^2^ weighted regression. The representative standard curve equation was

y = 0.024793x + 0.0456153(1)

and the correlation co-efficient (r) was ≥0.99 (r = 0.9988, *n* = 7).

The analyte response at the lower limit of quantification (LLOQ) was 20 ng/mL (%RSD = 6.5%, *n* = 5, S/N > 10), and the lower limit of detection (LLOD) was 10 ng/mL (*n* = 5, S/N > 3).

The inter- and intra-assay accuracy was 92.5–102.9% and 92.2–101.5%, respectively. The intra- and inter-assay precision was 0.8–6.5% and 0.4–5.4%, respectively. These results met the precision requirement of up to 15%, except for the LLOQ, where a value of ≤20% was observed. The accuracy was within the range of 85–115%, and the LLOQ would include the range of 80–120% (Table 1).

Pre-and post-extracted plasma standards containing 60, 1000, and 1600 ng/mL CPPU were analyzed, and the recovery was 82.5–93.1%. The absolute recovery of 200 ng/mL IS was 106.5% (Table 2). The peak area ratios of CPPU with the post-extraction blank sample to CPPU in the mobile phase verified the absence of significant matrix effects (MEs) in our method (Table 2).

CPPU samples were stable after storage at room temperature (20–24 °C) for 12 h, at −20 °C for 14 days, after at least three freeze-thaw cycles at −20 °C, and in the sample rack (4 °C) for 24 h (Table 3).

The accuracy and precision of CPPU detection under the corresponding dilution factors were within ±15%, indicating that the plasma samples were stable under the 10× dilution conditions. The residue was determined by measuring the blank plasma sample after detecting the upper limit of the standard curve, and the residual response measured in the blank sample was not more than 20% of the LLOQ, indicating that the residue met the requirements.

### 2.4. Application in a Comparative PK Study of Rats

**Figure 2:**
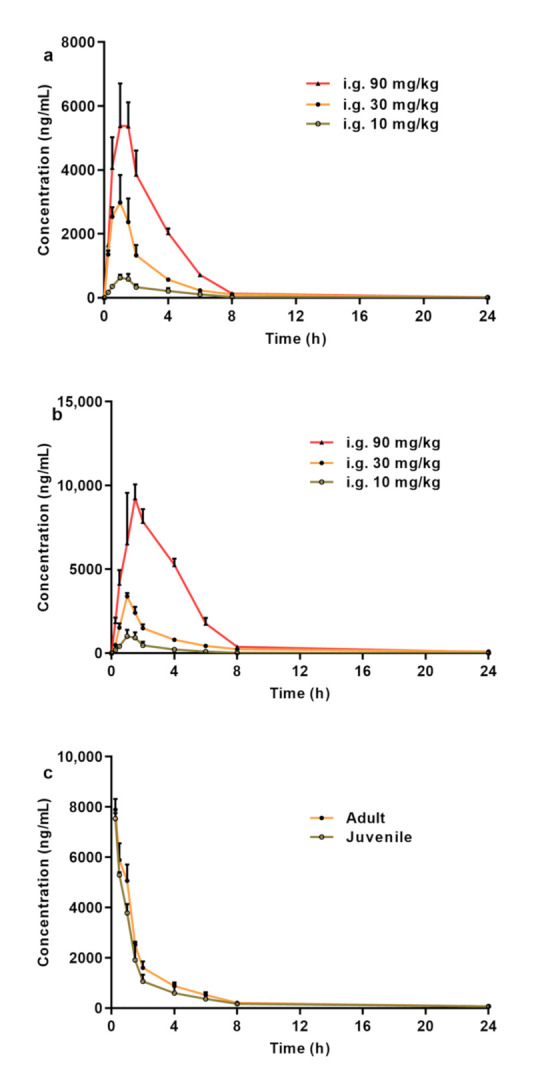
(**a**) Mean plasma concentration–time curves following the oral administration of CPPU in adult rats. (**b**) Mean plasma concentration–time curves following oral administration of CPPU in juvenile rats. (**c**) Mean plasma concentration–time curves following intravenous administration of CPPU in both adult and juvenile rats. CPPU—1-(2-chloro-4-pyridyl)-3-phenylurea, forchlorfenuron.

**Figure 3:**
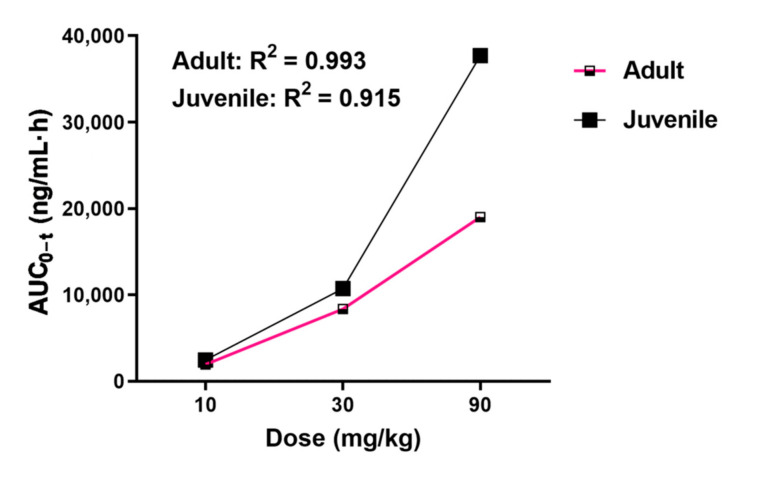
Area under the concentration-time curve from time “0” to the time of the last measurable concentration (AUC_0–t_) vs. CPPU dose: comparison between adult and juvenile rats. CPPU—1-(2-chloro-4-pyridyl)-3-phenylurea, forchlorfenuron.

In this study, a rapid, simple, and efficient method was developed for the determination of CPPU in rat plasma. Using this method, we performed a comparative PK study of CPPU in adult and juvenile rats. The mean plasma concentration-time curves obtained after the oral administration of CPPU to adult and juvenile rats are shown in Figure 2. Using DAS v.3.1 software, the non-compartment model was found to be the most rational PK model in both adult and juvenile rats. The correlation between AUC_0–t_ and dose is shown in Figure 3. The CPPU did not induce toxicity in rats at the administered doses, and the vital signs of the rats were normal. The PK parameters are shown in Table 4.

Both the AUC_0–t_ and maximum drug concentration (C_max_) in adult and juvenile rats treated with CPPU at 10–90 mg/kg increased in a dose-dependent manner. The AUC_0–t_ and F values in the juvenile rats were higher than those in the adult rats. The T_1/2_ in adult rats was similar to that in the juvenile rats. The mean residence time (MRT_0–t_) and AUC_0–t_ of juvenile rats in the gavage groups, except for the 10 mg/kg dose, were higher than those in adult rats (*p* < 0.001). Although the C_max_ of the 30 mg/kg dose group (juvenile rats) increased slightly, the C_max_ of the 10 and 90 mg/kg groups showed a significant increase. The plasma clearance (CLz) of CPPU in juvenile rats was slightly lower than that in adult rats. These results indicate that juvenile rats are more sensitive to CPPU.

According to the toxicology data issued by the United States Environmental Protection Agency (US EPA [19]), chronic and sub-chronic studies have indicated that CPPU is toxic to developing zebrafish, as determined during screening for developmental toxicity, with a half-maximal activity of 46 μM [20]. Another study found that the administration of high-dose CPPU to rats (115 mg/kg for males and 205 mg/kg for females) caused slight decreases in food intake and body weight of first (F1) and second (F2) filial generation animals [21]. Zhu et al. conducted a study on the developmental toxicity of CPPU administered to rats via gavage prenatally and postnatally. They found that CPPU may promote estradiol secretion, resulting in altered vaginal opening and first estrus time in prepubertal female rats [3]. These studies suggest that CPPU has potent effects on animal reproduction and development. While a few analytical methods for CPPU detection, such as high-performance liquid chromatography (HPLC) with ultraviolet detection [22], LC-MS/MS [13,22], and LC/time-of-flight/MS [23,24], few reports evaluating the PK of CPPU were published to date. We studied the PK characteristics of CPPU in adult and juvenile rats and found that AUC_0–t_ and absolute bioavailability were higher in juvenile than in adult rats, which may be due to metabolic differences.

## 3. Materials and Methods

### 3.1. Chemicals and Reagents

**Figure 4:**
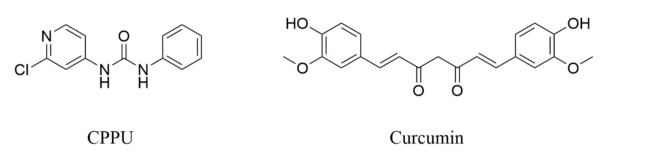
Structural formulas of 1-(2-chloro-4-pyridyl)-3-phenylurea (CPPU, forchlorfenuron), and curcumin.

CPPU (99% purity) was provided by Bio Basic Canada (Markham, Ont., Canada). Curcumin (IS; 98.7% purity) was purchased from the National Institutes for Food and Drug Control (Beijing, China). The structures of CPPU and IS are shown in Figure 4. Acetonitrile, methanol, and formic acid were provided by Merck (Darmstadt, Germany). All solvents were of chromatography grade. Purified water was purchased from ELGAPURELAB Ultra System (High Wycombe, UK).

### 3.2. Animals

Adult (6- to 8-week-old, weight: 200–220 g) and juvenile (3- to 4-week-old, weight: 80–100 g) Sprague-Dawley rats (equal numbers of males and females) were obtained from Vital River Laboratory Animal Technology Co. (Beijing, China). The animals were housed at an ambient temperature of 20–26 °C and humidity of 40–70%. The pressure between the room and corridor was greater than 10 Pa, with a ventilation over 15 times per hour, and a 12/12-h light/dark cycle (08:00–20:00). The animals were acclimatized to these conditions for one week and were fasted for at least 12 h before the experiments. The study was approved by the Institutional Animal Care and Use Committee of CDSER of Zhejiang University (IACUC-17-211).

### 3.3. Instrumentation and Chromatographic Conditions

A triple-quadrupole ultra-high-performance liquid chromatography (UPLC) system (Xevo, Waters Corp., Milford, MA, USA) was used for CPPU analysis. Considering the properties of CPPU and large sample volumes, various UPLC columns with different pore diameters were investigated. Based on our optimization experiments, ACQUITY UPLC BEHC18 column (2.1 mm × 50 mm, 1.7 μm, Waters) was selected and the analysis temperature was set at 30 °C. Acetonitrile/water and methanol/water binary solvent systems using different buffers, such as ammonium formate and formic acid, were tested; acetonitrile (B) and water containing 0.1% formic acid (B) was eventually selected as the mobile phase at a flow rate of 0.3 mL/min. Gradient elution was performed as follows: 0–2 min, 10–95% B; 2–3.5 min, 95% B; 3.5–4 min, 95–10% B; and 4–5 min, 10% B.

CPPU and curcumin were detected using the positive multiple reaction monitoring mode. CPPU ions were detected at *m/z* 248.02→93.07, and curcumin ions were detected at *m/z* 369.09→176.99. The MS operating parameters were optimized as follows: source temperature, 150 °C; desolvation temperature, 500 °C; capillary voltage, 3 kV; collision gas flow, 0.15 mL/min; cone gas flow, 150 L/h; desolvation gas (nitrogen) flow, 650 L/h; and collision energy, 36 and 22 V for CPPU and curcumin, respectively. Data processing was performed using the MassLynx v.4.1 software (Waters Corp).

### 3.4. Preparation of Standard and Quality Control Samples

Stock solutions of CPPU (1 mg/mL) and curcumin (1 mg/mL) were separately prepared in methanol. Calibration standard samples for CPPU were diluted with methanol to obtain concentrations of 40, 20, 10, 4, 2, 1, and 0.4 μg/mL. Curcumin standard solution was prepared at 200 ng/mL with methanol. All standard solutions were kept at −20 °C until use.

Each blank rat plasma sample (95 μL) was spiked with different concentrations of CPPU (5 μL) and divided into two parts after mixing. One part (50 μL) was used for the calibration standard samples at concentrations of 20, 50, 100, 200, 500, 1000, and 2000 ng/mL. The other was kept as a spare portion. The quality control CPPU samples (20, 60, 1000, and 1600 ng/mL) were prepared in an identical manner.

### 3.5. Sample Preparation

Methanol precipitation and ethyl acetate liquid-liquid extraction methods were investigated based on the compound properties. Subsequently, methanol precipitation was selected, and a good extraction recovery rate was achieved. Proteins in rat plasma samples were precipitated using three volumes of methanol. Thereafter, 50 µL of plasma was pretreated in the same way (three volumes of methanol, in addition to curcumin), vortexed for 10 min, and centrifuged at 12,000× *g* for 10 min. The supernatant (10 μL) was analyzed using LC-MS/MS. The samples for method validation were pretreated in the same way (three volumes of methanol, in addition to curcumin).

### 3.6. Method Validation

Method validation was conducted following the Bioanalytical Method Validation Guidance for Industry [24]. The following items were investigated: specificity, linearity, precision and accuracy, LLOQ, recovery rate, ME, and dilution reliability.

The chromatograms of six individual sources of blanks were applied to assess specificity. The method was considered specific if the response attributable to interfering components was not more than 20% of the analyte response at the LLOQ and not more than 5% of the IS response in the LLOQ sample.

Plasma samples of the standard curve were spiked using seven CPPU concentrations (20, 50, 100, 200, 500, 1000, and 2000 ng/mL). The peak area ratio of CPPU to curcumin was calculated and the standard curve was drawn by plotting the peak area ratio vs. analyte concentration. LLOQ was determined as the minimum concentration of the calibration curve. A signal-to-noise ratio of 3 was used to determine LLOD.

Accuracy and precision were evaluated based on the performance of Quality Control (QC) samples within each run and between different runs. Three batches were continuously detected to investigate the intra-batch and inter-batch precision. The intra-batch and inter-batch precision must be <15%, with an accuracy ranging between 85% and 115%, except for LLOQ (no more than 20% or within the range of 80–120%).

Recovery was computed by comparing the peak area ratios of QC samples (at different concentrations) with those of spiked, post-extraction blank samples. The acceptance criterion was a ≤30% difference between the QC concentration recoveries.

The ME was investigated by contrasting the peak responses of pure solution samples at three QC concentrations of CPPU and at a single concentration of IS. The acceptance criterion was set at no more than 15% for %RSD.

Sample stability was determined at three concentrations (60, 1000, and 1600 ng/mL, *n* = 3 per concentration) under different test conditions: (1) short-term stability at room temperature (15–30 °C) for 12 h; (2) long-term stability at −20 °C for 14 days; (3) post-preparation stability in the autosampler at 4° C for 24 h; and (4) after three freeze-thaw cycles at −20° C.

Some plasma sample concentrations were out of range of the standard curve during sample detection. These samples were diluted and retested. The dilution factor of CPPU was set to 10 according to the linear range. The accuracy and precision were supposed to be within 15%.

For residue verification, residues were determined by examining the upper limit of the standard curves and blank plasma samples. The responses measured using the blank samples were supposed to be <20% of the LLOQ.

### 3.7. PK Study

The national long-term dietary estimated daily intake safety evaluation study of the Australian Bureau of Chemical Safety showed that the no observed effect level (NOEL) of CPPU is 7 mg/kg/person/day [25]. According to the conversion of animal and human doses, the NOEL of rats is therefore ~40 mg/kg. The CPPU doses for rats were consequently set to 10, 30, and 90 mg/kg. Adult rats (*n* = 24; *n* = 6 per group) were fasted for at least 12 h and randomly divided into three treatment groups for oral administration of 10, 30, or 90 mg/kg CPPU (in 0.5% carboxymethyl cellulose), and another treatment group received intravenous administration of 5 mg/kg CPPU (in saline). The juvenile rats (*n* = 24; *n* = 6 per group) were also randomly divided into four groups following the same dosing regimen. CPPU was suspended in 0.5% carboxymethyl cellulose at 1, 3, and 9 mg/mL for oral administration and in saline at 0.5 mg/mL for intravenous administration. Approximately 100 μL blood samples were withdrawn into heparinized tubes using the tail bleed method before the administration as well as at 0.25, 0.5, 1, 1.5, 2, 4, 6, 8, and 24 h following administration. The blood samples were immediately centrifuged at 1000× *g* for 10 min, and the plasma samples were frozen at −20° C until analysis.

The animal studies were approved by the Animal Research Committee at Zhejiang University (Approval number IACUC-17-211), and all experimental protocols were conducted in accordance with the institutional guidelines. DAS v.3.1 software (DAS, Shanghai, China) was used to calculate and analyze the PK parameters such as AUC_0–t_, C_max_, T_1/2_, MRT, and CL. SPSS Statistics v19.0 (IBM, Armonk, NY, USA) was used to perform statistical tests for the data comparisons. A test for homogeneity of variance was performed. If the variance was uniform (*p* > 0.05), a one-way analysis of variance was performed. If the variance was not uniform (*p* < 0.05), a non-parametric test (Kruskal-Wallis H test) was performed. The summary table report lists the results for the PK parameters AUC_0–t_ and C_max_ at significance levels of 0.05, 0.01, and 0.001 after comparison.

## 4. Conclusions

In conclusion, a rapid and simple LC-MS/MS method for CPPU detection was developed and validated. We then employed this method to investigate the PK characteristics of CPPU in adult and juvenile rats. The AUC_0–t_ and C_max_ of both adult and juvenile rats treated with CPPU at 10–90 mg/kg increased in a dose-dependent manner. The T_1/2_ in adult rats was similar to that in juvenile rats. The AUC_0–t_ and absolute bioavailability were higher in the latter. These results indicated that juvenile rats were more sensitive to CPPU, which provides an experimental basis for the study of pesticide exposure in children. We hope that the current findings provide insights into the potential safety risks of CPPU, particularly in children.

## Figures and Tables

**Table 1:** Inter- and intra-assay accuracy and precision of CPPU detection in rat plasma (*n* = 5).

Concentration of CPPU (ng/mL)	Intra-Assay			Inter-Assay		
	Mean ± SD	Precision (%)	Accuracy (%)	Mean ± SD	Precision (%)	Accuracy (%)
20	20.6 ± 1.3	6.5	102.9	20.2 ± 1.1	5.4	101.2
60	61.4 ± 1.5	2.5	102.3	60.9 ± 0.5	0.9	101.5
1000	933.3 ± 18	1.9	93.3	952.8 ± 17.4	1.8	95.3
1600	1478.9± 12.2	0.8	92.5	1475.5 ± 6.4	0.4	92.2

CPPU, 1-(2-chloro-4-pyridyl)-3-phenylurea, forchlorfenuron.

**Table 2:** Matrix effect and recovery of CPPU and IS (*n* = 6).

Substance	Concentration of CPPU (ng/mL)	RE (%)	ME (%)
CPPU	60	85.7 ± 0.4	90.5 ± 1.2
	1000	82.5 ± 4.9	98.1 ± 3.2
	1600	93.1 ± 1.3	96.4 ± 1.1
IS	200	106.5 ± 5.3	108.3 ± 5.2

ME—matrix effect; RE—recovery; IS—internal standard (curcumin); CPPU—1-(2-chloro-4-pyridyl)-3-phenylurea, forchlorfenuron.

**Table 3:** Stability of CPPU (*n* = 3).

Spiked Samples (ng/mL)	12 h (Benchtop)		24 h (Autosampler)		14 Days (at −20 °C)		Three Freeze-Thaw Cycles	
	Mean ± SD	Accuracy (%)	Mean ± SD	Accuracy (%)	Mean ± SD	Accuracy (%)	Mean ± SD	Accuracy (%)
60	57.5 ± 1.7	95.9	58.5 ± 1.3	97.5	61.6 ± 0.9	102.7	61.4 ± 1.6	101.7
1000	974.6 ± 16.7	97.5	970.6 ± 8.4	97.1	1042.7 ± 23.1	104.3	1030.7 ± 12.1	103.1
1600	1564.9 ± 23.7	97.8	1546.3 ± 27.3	96.6	1664.4 ± 17.5	104.0	1632.9 ± 8.9	102.1

CPPU—1-(2-chloro-4-pyridyl)-3-phenylurea, forchlorfenuron.

**Table 4:** Pharmacokinetic (PK) parameters of CPPU.

Animals	Dose (mg/kg)	C_max_ (ng/mL)	T_1/2_ (h)	CLz (L/h/kg)	MRT_0–t_ (h)	AUC_0–t_ (ng /mL·h)	AUC_0–∞_ (ng /mL·h)	F (%)
Adult rats	10 (p.o.)	711.1 ± 70.4	2.6 ± 1.1	5.0 ± 1.3	3.0 ± 0.6	1935.7 ± 361.5	2108.3 ± 553.3	6.1
	30 (p.o.)	3266.3 ± 50.2	2.4 ± 1.1	3.4 ± 0.7	3.1 ± 0.6	9294.4 ± 862.0	9310.6 ± 867.7	9.8
	90 (p.o.)	6273.3 ± 133.1	2.5 ± 0.8	4.6 ± 0.2	2.9 ± 0.1	18,915.9 ± 622.7	18,967.7 ± 643.2	6.6
	5 (i.v.)	7977.4 ± 143.7	1.8 ± 0.1	0.3 ± 0.01	3.0 ± 0.1	15,851.6 ± 351.9	15,852.4 ± 352.8	-
Juvenile rats	10 (p.o.)	1148.9 ± 145.1 **	2.6 ± 1.1	3.8 ± 0.9	2.6 ± 0.3	2745.5 ± 632.7	2753.6 ± 608.1	10.2
	30 (p.o.)	3386.1 ± 195.5	2.3 ± 0.2	2.8 ± 0.2	4.9 ± 0.1 ***	10,713.5 ± 559.5 ***	10,720.4 ± 563.4 ***	13.3
	90 (p.o.)	9805.7 ± 77.5 ***	2.5 ± 0.1	2.5 ± 0.1	3.3 ± 0.1 ***	36,442.7 ± 1086.1 ***	36,513.9 ± 1085.8 ***	15.1
	5 (i.v.)	7571.2 ± 183.1	1.7 ± 0.4	0.3 ± 0.04	2.9 ± 0.3	13,470.0 ± 1281.6	13,471.3 ± 1280.4	-

Data are presented as the mean ±SD (*n* = 6). i.v.—intravenous; p.o.—per oral; C_max_—the maximum drug concentration; T_1/2_—the time taken for half the initial drug concentration to be eliminated; CLz—plasma clearance; MRT_0–t_—mean residence time; AUC_0–t_—the area under the plasma drug concentration–time curve from 0 to 24 h, the final time point sampled; AUC_0–∞_—the area under the plasma drug concentration–time curve from 0 to infinity; comparison between the doses in adult and juvenile rats; F—bioavailability, CPPU—1-(2-chloro-4-pyridyl)-3-phenylurea, forchlorfenuron. ** *p* < 0.01 and *** *p* < 0.001.

## Data Availability

The data that support the findings of this study are available on request from the corresponding author. The data are not publicly available due to privacy or ethical restrictions.

## Sample Availability

Sample of the compounds are not available from the authors.
